# A reproducible and quantifiable model of choroidal neovascularization induced by VEGF A^165^ after subretinal adenoviral gene transfer in the rabbit

**Published:** 2008-07-30

**Authors:** Sylvie Julien, Florian Kreppel, Susanne Beck, Peter Heiduschka, Veronica Brito, Sven Schnichels, Stefan Kochanek, Ulrich Schraermeyer

**Affiliations:** 1 Section of Experimental Vitreoretinal Surgery, University Eye Hospital of Tuebingen, Germany; 2 Division of Gene Therapy, University of Ulm, Germany; 3 Retinal Diagnostics Research Group, University Eye Hospital of Tuebingen, Germany

## Abstract

**Purpose:**

To determine the effects of the vascular endothelial growth factor (VEGF)-A^165^ delivered using a high capacity adenoviral vector (HC Ad.VEGF-A) on vascular growth and pathological changes in the rabbit eye. To combine different detection methods of VEGF-A^165^ overexpression-induced neovascularization in the rabbit.

**Methods:**

HC Ad.VEGF-A^165^ was constructed and injected at 5x10^6^ infectious units (iu) into the subretinal space of rabbit eyes. Two and four weeks postinjection, the development of neovascularization and the expression of HC Ad-transduced VEGF-A^165^ protein were followed up in vivo by scanning laser ophthalmoscopy, fluorescein and indocyanine green angiographies and ex vivo by electron microscopy and immunohistochemistry

**Results:**

We observed a choroidal neovascularization (CNV) with leakage in 83% of the rabbit eyes. Our findings present clear indications that there is a significant effect on the endothelial cells of the choriocapillaris after subretinal transduction of the retinal pigment epithelium (RPE) with VEGF-A^165^ vector. The choroidal endothelial cells were activated, adherent junctions opened, and the fenestration was minimized, while the extracellular matrix localized between the RPE and the endothelium of the choriocapillaris was enlarged toward the lumen of the vessels, inducing a deep invagination of the endothelial cells into the vessel lumen. They also proliferated and formed pathological vessels in the subretinal space. Moreover,there was an increased expression of basic fibroblast growth factor and VEGF-A accompanied by macrophage stimulation, retinal edema, and photoreceptor loss.

**Conclusions:**

This is the first model of VEGF-induced CNV in the rabbit in which the pathological events following overexpression of VEGF by RPE cells have been described in detail. Many of the features of our experimental CNV resemble those observed clinically in patients having wet age-related macular degeneration.

## Introduction

Pathological angiogenesis is a major problem in many ocular diseases. The most important angioproliferative diseases in ocular tissues include diabetic proliferative retinopathy, age-related macular degeneration (AMD) in adults, and retinopathy of prematurity in infants. These diseases are the most common causes for blindness in developed countries [1]. Choroidal neovascularization (CNV) is an important component of subsequent vision loss in neovascular AMD [2]. In patients with the exudative or “wet” form of the disease, choroidal blood vessels grow through Bruch’s membrane into the subretinal space, followed by leakage and accumulation of serum or blood beneath the retinal pigment epithelium (RPE), leading to insult of the retina and rapid vision loss [3]. Although the molecular basis of AMD is not well understood, several growth factors have been implicated in the disease process, including basic fibroblast growth factor (bFGF/FGF-2) [4], transforming growth factor (TGF)-β [5], and vascular endothelial growth factor (VEGF) [4,6,7]. Several lines of evidence suggest that VEGF is a major stimulator of CNV in AMD, including an observed increase of VEGF in the RPE of maculae from patients with AMD [8], increase of VEGF expression in the laser model of CNV in monkey [9] and rat [10], and induction of CNV in ectopically delivered VEGF cDNA to the RPE of rats [11-13] and nonhuman primates [14]. VEGF is a secreted peptide that has five homodimeric variants formed by alternative splicing: VEGF-A, VEGF-B, VEGF-C, VEGF-D, and placenta growth factor (PIGF). These differ in their ability to bind to VEGF receptors that are primarily expressed in endothelial cells. Of these variants, the 165-amino-acid-long VEGF-A^165^ is the most commonly expressed isoform in the ischemic retina [15]. Because of cumulative evidence for the involvement of VEGF in AMD, several clinical trials targeting the VEGF pathway are under way [16]; however the precise role of VEGF-A in the pathogenesis of CNV still remains unclear. High levels of VEGF have been found in excised CNV, but overexpression of VEGF-A specifically in RPE cells failed to induce CNV [17]. Moreover, antiangiogenic roles of VEGF-A have been described [18].

The lack of optimal therapy is partially due to the unavailability of appropriate large animal models for the testing of new treatment options for ocular angioproliferative diseases. Existing animal models of CNV present several problems: they are hard to reproduce, they are inefficient, and the CNV created is not sustainable. The purpose of this study was to develop an efficient, reliable model of CNV to facilitate the study of antiangiogenic and antiproliferative therapies for ocular diseases in the future. We have developed a new model for CNV by injection of a high-capacity adenovirus vector encoding for VEGF-A^165^ (HC Ad.VEGF) into the subretinal space of rabbits. Gene transduction with vector expressing VEGF is a well established model as shown in the past [13]. Human adenoviruses of serotype 5 are the preferred vehicle for in vitro and in vivo gene transfer because of their low pathogenicity. For the induction of CNV, a high-capacity “gutless” adenoviral vector with the expression cassette for the human VEGF-A^165^ (hVEGF promoter), based on plasmid pBLAST was used. This type of vector is devoid of viral coding sequences and allows for long-term in vivo gene expression in resting cell types [19,20].

Moreover, for the first time we have combined detection methods of VEGF-A^165^ overexpression-induced neovascularization in mammals: in vivo by scanning laser ophthalmoscopy (SLO), fluorescein angiography (FA), and indocyanine green angiography (ICG) and ex vivo by electron microscopy and immunohistochemistry. This has made it possible to elucidate the unsettled question of whether, after VEGF overexpression, vascular leakages are induced by pathological fenestration in new or existing vessels, by loss of endothelial cells, or by opening adherent junctions.

## Methods

### Generation of pHC Ad.VEGF-A^165^, pHC Ad.FK7, and pAd. empty

The vectors were produced by one of the authors (S.K.). To generate pHC Ad.VEGF-A^165^, the expression cassette for human VEGF-A^165^ was excised from the plasmid pBLAST49-hVEGF (Invivogen, San Diego, CA) by PvuI and SwaI, blunt-ended with T4 polymerase, and subcloned into the unique SwaI site of pSTK129. Expression of VEGF-A^165^ in this expression cassette is driven by a hybrid EF1α/HTLV hybrid promoter and a Simian virus 40 polyadenylation signal. pSTK129 is a plasmid used for the generation of HC Ad vectors and contains the left terminus of adenovirus serotype 5 (nucleotides 1 through 440), a 20 kb DNA fragment derived from the human hypoxanthine-guanine phosphoribosyltransferase (HPRT) locus HUMHPRTB (gene map positions 1777–21729), a 6.5 kB human fragment of C346 (locus HUMDXS455A, cosmid map positions 10205–16750), and the right terminus of adenovirus serotype 5 (nt 35818–35935). The SwaI site used to insert the VEGF expression cassette is located within the HPRT stuffer region. Generation of HC-AdFK7 has been serially amplified on 293cre66 cells and purified by double CsCl density gradient centrifugation as described in detail elsewhere [19]. pAd. empty vector is an E1-deleted first-generation adenovirus vector without transgene expression cassette.

### Generation of high titer purified HC Ad vector stocks

The plasmids pHC Ad.VEGF-A^165^ and pHC A.FK7 were linearized by PmeI and transduced into 293cre66 cells, which were subsequently infected with AdLC8cLuc at a multiplicity of infection (MOI) of 5 [21]. Vectors were serially amplified with 5 MOI helper virus on 293cre66 cells as described in detail elsewhere [20] and purified by discontinous and continous CsCl density gradient centrifugation. Purified vectors were desalted by PD-10 columns (Amersham, Freiburg, Germany), and the physical titer, infectious titer, and helper virus contamination were determined by a DNA-based slot-blot assay [22]. The vector preparations used in this study had a helper virus contamination between 2%–4%.

### Cell culture

The ARPE-19 cell line was obtained from the American Type Culture Collection (Manassas, VA), human umbilical vein endothelial cells (HUVEC) from Provitro (Berlin, Germany). ARPE-19 cells were maintained in Dulbecco’s modified Eagle’s medium (DMEM) with 10% fetal bovine serum. HUVEC cell medium was supplemented with 22.50 ng/ml of heparin and 5 ng/ml of endothelial cell growth factor.

### In vitro detection of VEGF-A

ARPE-19 cells were seeded in six-well plates at 10^6^ cells/well. HC Ad.VEGF-A^165^ was added at a MOI of 20 and left for 48 h. The media were then replaced and the cells left for an additional seven days. Supernatants (SN) were then collected, and secreted VEGF-A was detected by sandwich ELISAs (DuoSet ELISA, R&D Systems, Minneapolis, MN). Supernatants from ARPE-19 cells not transduced with HC Ad.VEGF-A^165^ were used as control.

### Effects of ARPE 19-produced VEGF-A^165^ on the function of human umbilical vein endothelial cells

#### Human umbilical vein endothelial cell proliferation assay

To analyze proliferation, we collected ARPE-19 supernatants. Undiluted supernatants or supernatants diluted 1:10, 1:100, or 1:1000 were added to HUVECs that were plated in 24 well plates at 5x10^3^ cells/well. We used a commercial VEGF-A^165^ (R&D Systems) in increasing dilutions (from 50 ng/ml to 0.05 ng/ml) as a positive control. Three days later, proliferation of the endothelial cells was measured by incubating at 37 °C in WST1 (Roche, Mannheim, Germany) for 4 h. The test was performed in an endothelial cell growth medium advanced (Provitro, Berlin, Germany) without endogenous VEGF and bFGF. The OD was measured at 450 nm.

#### Human umbilical vein endothelial cell migration assay

To analyze migration, we incubated 5x10^4^ HUVEC per insert in Millicell 24 well hanging cell culture insert, PET 8 μm (Millipore, Bedford, MA) in the same medium used for the proliferation test with or without the ARPE-19 supernatant (undiluted) for 6 h at 37 °C. As a positive control, we used the commercial VEGF-A^165^ (R&D Systems) at the same dilutions that we used for the proliferation test. Migrated cells were visualized by the nuclear fluorescence dye 4’,6-diamidino-2-phenylindole (DAPI; Alexis, Grünberg, Germany) and were counted in three random fields per insert.

### Animals

Adult Chinchilla Bastard rabbits, weighing 2–2.5 kg, were obtained from Charles River, Sulzfeld, Germany. The ad libitum-fed animals were treated in compliance with the ARVO Statement for the Use of Animals in Ophthalmic and Vision Research.

### Subretinal injections

The rabbits were anesthetized by an intramuscular injection of 35 mg/kg ketamine hydrochloride and 5 mg/kg xylazine hydrochloride. Their pupils were dilated with 0.5% tropicamide and 2.5% phenylephrine eye drops. The ocular surface was additionally anesthetized with topical instillation of 0.5% proparacaine hydrochloride eye drops.

Using an operating microscope, we made a 3 mm paralimbal conjunctival incision in the supranasal quadrant, and performed a sclerotomy 2 mm posterior to the corneal limbus. A 30-gauge needle attached to a Hamilton syringe (Hamilton Co., Reno, NV) was inserted through the sclerotomy site into the vitreous cavity. High-capacity adenoviral vector encoding VEGF-A^165^ was injected into the subretinal space of rabbits at 5x10^6^ infectious units. We chose to inject the HC Ad.VEGF-A at this optimal dose based on results from our previous study about the long-term transgene expression in the RPE after gene transfer with a high-capacity adenoviral vector [19]. The injection resulted in a locally restricted subretinal bleb. In the control animals, 20 μl of either PBS or HC-AdFK7 (5x10^6^ infectious units) were injected into the subretinal space at a similar location. Topical antibiotic was applied after the injection.

### Follow-up examination

Follow-up procedures included scanning laser ophthalmoscopy (SLO), fluorescein angiography (FA) and indocyanine green angiography (ICG). We used a Heidelberg Engineering HRA I SLO (Heidelberg Engineering, Dossenheim, Germany) modified for the use with animals [23]. The HRA I features the two argon wavelengths (488 nm and 514 nm) in the short wavelength range and two infrared diode lasers (795 nm and 830 nm) in the long wavelength range. The 488 and 795 nm lasers were used for FA and ICG angiography. Two device settings for the field of view were used: 20° for fundus overview, and 10° for detailed view. Light of shorter wavelengths is generally more strongly attenuated by ocular structures than that of longer ones; RPE and choroids especially are, in addition to the photoreceptors, the main absorbing tissues for visible light due to their melanin content. In pigmented animals, the short-wavelength lasers provide the highest quality images of the retina, but cannot penetrate the RPE and choroids. In contrast, the infrared lasers show less retinal detail but reveal choroidal structures down to the sclera. Accordingly, FA provides the most detailed images of retinal capillaries, whereas ICG adds information about choroidal vessels but shows less retinal vasculature detail. An important difference between the dyes is their affinity to (large) plasma proteins. ICG is bound to such proteins to more than 98%, whereas fluorescein is only bound to about 60%–80%. As a consequence, ICG diffuses only very slowly out of the vascular lumen even if vessels are fenestrated, whereas fluorescein tends to leak rapidly [24].

Prior to examination, the animals were anesthetized as described in the previous section, and their pupils were dilated with 0.5% tropicamide and 2.5% phenylephrine eye drops. A contact lens (Geuder, Heidelberg, Germany) was used to avoid dehydration of the cornea. FA and ICG were performed using an intravenous injection of a mixture of 37.5 mg/kg bodyweight fluorescein-Na (University Pharmacy, University of Tuebingen, Germany), and 25 mg/kg bodyweight ICG (ICG-Pulsion, Pulsion Medical Systems AG, Munich, Germany).

SLO, FA, and ICG were performed in all animals at postoperative weeks two and four, focusing on the injection site area. The rabbits were sacrificed by an intracardial injection of 5 ml T61 (Intervet, Unterschleissheim, Germany) immediately following ophthalmoscopy. All eyes were enucleated and were processed either for examination by electron microscopy or by immunohistochemistry.

### Quantification of the fluorescence intensities

For quantification, images of the fluorescein angiography from six animals (three for the control HC Ad.FK7 and three for the HC Ad.VEGF-A) were analyzed with the Openlab software (Improvision, Tuebingen, Germany). In all animals, the light intensity at the lesion site and at an area of approximately the same size near the lesion site, which served as a background control, was measured. The Maximum value (white) was 255, and the minimum value (black) was 0. The intensity of the background control was then subtracted from the intensity of the lesion site (Δ_light intensity_). The maximum deviation of Δ_light intensity_ is indicated by bars.

### Light and electron microscopy

The eyes were cleaned of orbital tissue and, after removal of the cornea, they were fixed overnight at 4 °C in 2% glutaraldehyde in 0.1 M cacodylate buffer (pH 7.4) containing 100 mM sucrose. After washing with cacodylate buffer, areas of interest in flat mount preparations were excised and post-fixed with 1% osmium tetroxide in 0.1 M cacodylate buffer at room temperature for 1 h. Dehydration was then started by a series of 10-min incubations in 30%, 50%, and 70% ethanol. The samples were stained with saturated uranyl acetate. Dehydration was continued by incubations in 70%, 80%, 96% ethanol (10 min each), absolute ethanol (two times for 15 min each), and propylene oxide (two times for 15 min each). The samples were then embedded in Epon (SPI-Pon™812 Epoxy Embedding Kit, SPI supplies, West Chester, PA). Semithin sections were stained with toluidine blue and examined by light microscopy (Axioplan2 imaging®, Zeiss, Göttingen, Germany). For electron microscopy the sections were cut ultrathin, stained with uranyl acetate and lead citrate and observed using an electron microscope (model 902 A; Carl Zeiss).

### Immunohistochemistry

The eyes were fixed in formalin, embedded in paraffin wax, cut to 5 μm sections, and deparaffinized according to standard procedures. The eyes were first stained with hematoxylin and eosin to locate the areas of interest. The following antibodies were used for the detection of immunoreactivity, respectively: 1:500 mouse monoclonal anti-human VEGF-A (C-1) antibody (sc-7269; Santa Cruz Biotechnology, Santa Cruz, CA), 1:2000 biotinylated lycopersicon esculentum lectin and 1:2000 fluorescein lycopersicon esculentum (tomato) lectin (Vector Laboratories, Burlingame, CA) for endothelial cells, 1:50 rabbit polyclonal anti-Ki67 antibody (ab833; Biozol, Eching, Germany) for proliferating cells, 1:2000 sheep antirabbit albumin (Bethyl Laboratories, Montgomery, TX), 1:50 mouse monoclonal anti-FGF/anti-FGF-2 (Upstate, clone bFM-2, Lake Placid, NY), and 1:50 mouse monoclonal antirabbit macrophages (RAM-11; MO633; Dako Cytomation, Carpinteria, CA). Stained retinal sections were embedded in FluorSave (Calbiochem, Darmstadt, Germany) and inspected using a fluorescent microscope.

### In vivo detection of VEGF-A

VEGF-A production was analyzed by performing ELISA (human VEGF-A assay kit, Quantikine; R&D Systems) on vitreous samples two and four weeks after transduction of RPE cells with HC Ad. VEGF-A (n=4), with HC Ad. EGFP (n=2), and in non operated rabbit (n=2). Duplicate measurements were obtained from all samples. Serial dilutions of recombinant human VEGF-A were included in all assays as a standard.

## Results

### In vitro quantification of HC Ad.VEGF-A^165^

ARPE-19 cells transduced with HC Ad.VEGF-A^165^ produced large amounts of VEGF-A^165^. ELISA was performed nine days after the transduction of the ARPE-19 cells, and the concentration of VEGF-A^165^ was 162±15 ng/ml. No secretion of VEGF-A^165^ was obtained in the nontransduced ARPE-19 cells.

### Biologic activity of HC Ad.VEGF-A^165^

**Figure 1:**
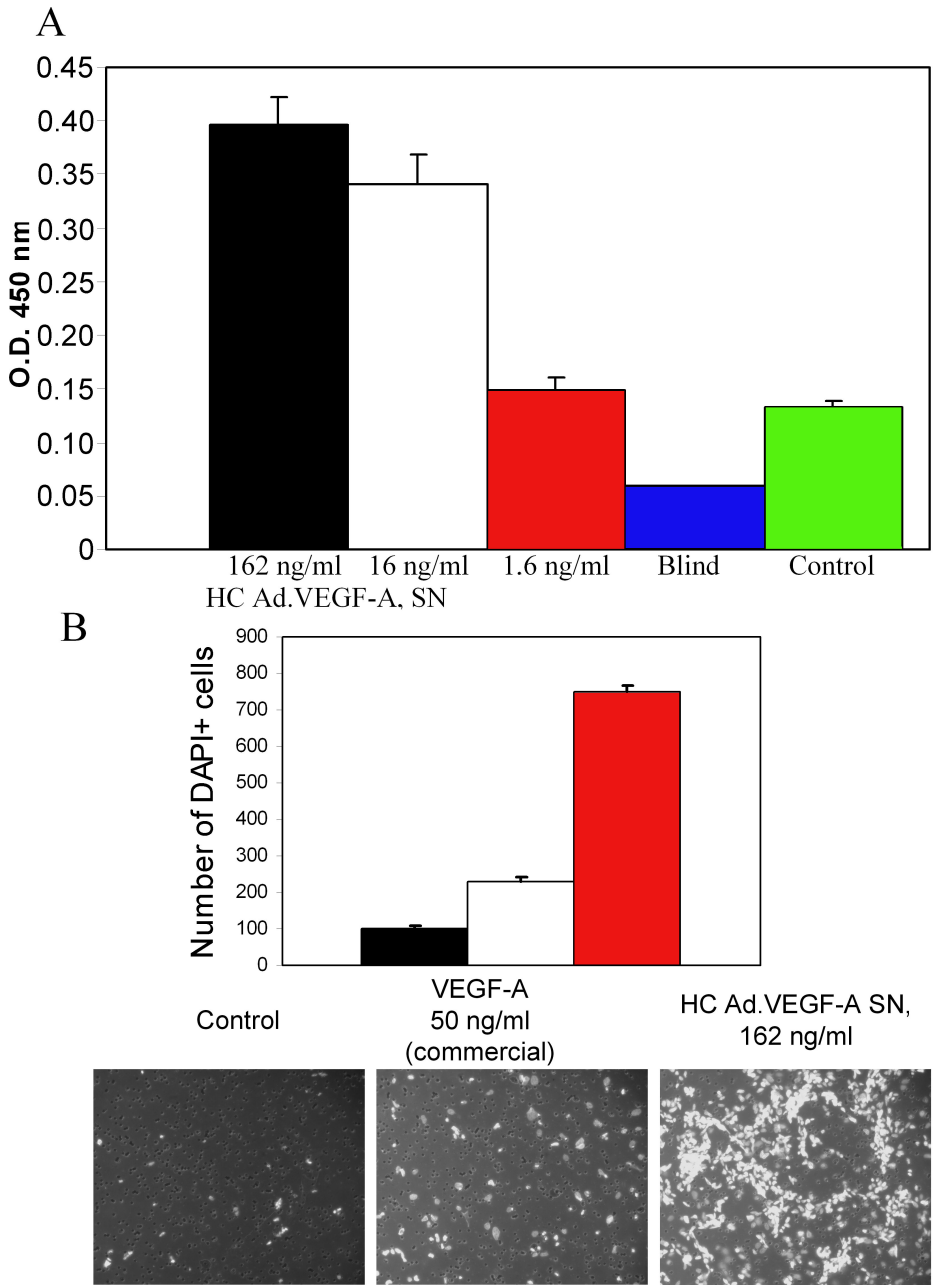
In vitro biological activity of high capacity adenovirus vector encoding for vascular endothelial growth factor A^165^ (HC Ad.VEGF-A^165^) on human umbilical vascular endothelial cells (HUVEC). **A:** HUVEC proliferation was measured in response to supernatants (SN) from ARPE-19 cells transduced with HC Ad.VEGF-A^165^ (MOI 20) with vascular endothelial growth factor (VEGF) protein concentrations as indicated. Blind means without HUVEC cells and control refers to medium without VEGF and bFGF. **B:** HUVEC migration was quantified in response to ARPE-19 cell supernatant (VEGF concentration 162 ng/ml), or commercial VEGF-A as a positive control. Each experiment was performed in triplicate and repeated on at least three occasions.

To test the biologic activity of VEGF-A^165^ produced by HC Ad.VEGF-A^165^ transduced ARPE-19 cells at a MOI of 20, we performed HUVEC proliferation and migration assays. For the proliferation test, the supernatants from transduced ARPE-19 cells were added to the HUVEC cells in increasing dilutions. Three days later, the relative proliferation of HUVEC cells in the presence or absence of VEGF-A^165^ was determined. The HUVEC cells proliferated in a VEGF-A concentration-dependent manner compared to the controls (blind and medium without VEGF and bFGF). However, at a 1:100 dilution (1.6 ng/ml), the ARPE-19 cells transduced with HC Ad.VEGF-A did not produce sufficient VEGF-A to still exert a proliferative effect (Figure 1A). As shown in Figure 1B, unstimulated HUVEC cells (control: medium without VEGF and bFGF in the lower chambers) exhibited minimal migration: after 6 h, only 100±5 cells per field had migrated to the undersurface of the filters. In contrast, when supernatants (162 ng/ml VEGF-A) from transduced ARPE-19 cells or a commercial VEGF-A (50 ng/ml) were added to the lower chambers, extensive and VEGF-A concentration-dependent HUVEC cells migration was observed (749±19 cells versus 228±12 cells per field).

### In vivo quantification of HC Ad.VEGF-A^165^

To estimate at which levels human VEGF-A is produced in vivo during the time of the follow-up examinations, an ELISA testing the amount of human VEGF-A in the vitreous was performed. In control eyes (not injected or after injection of HC Ad. EGFP), human VEGF-A could not be detected. In all VEGF-A transduced eyes, human VEGF-A was present in a stable concentration in the vitreous (67±12 pg/ml and 40±10 pg/ml, respectively) two and four weeks after the gene transfer.

### Fluorescence angiography in scanning laser ophthalmoscopy

**Figure 2:**
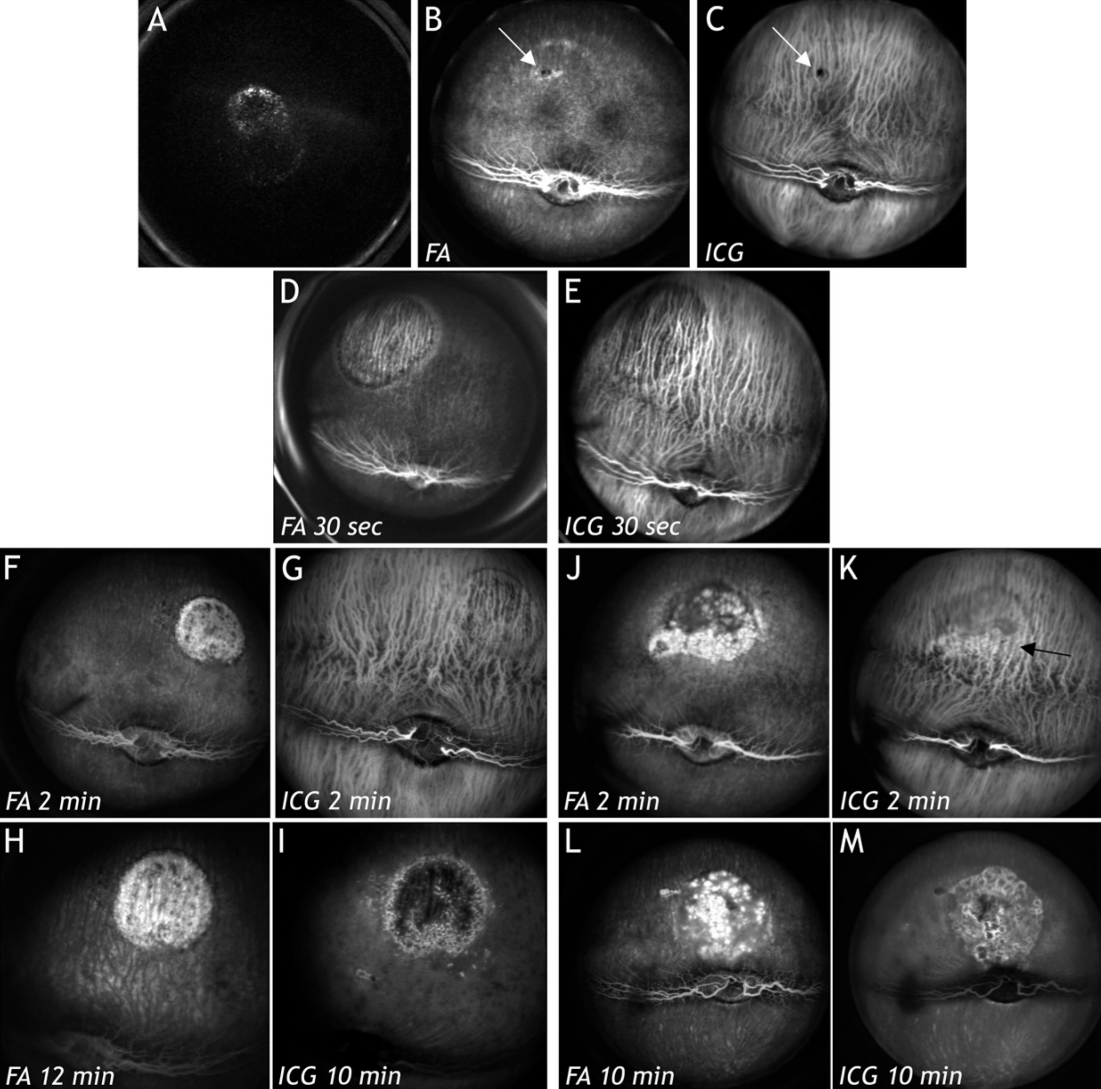
In vivo examination of the neovascularization development in rabbit eyes. **A:** After transduction with HC Ad.EGFP FK7, retinal pigment epithelium cells expressed green fluorescent protein in vivo. Fluorescein angiography (FA; **B**) or indocyanine green angiography (ICG; **C**) did not show blood vessel leakage or abnormality. The injection site is marked by white arrows. **D** and **E**: After transduction with Ad. Empty, which is toxic to the RPE, FA (**D**) and ICG (**E**) showed the same image representing a typical window effect. Two weeks (**F-I**) and four weeks (**J-M**) after transduction with high capacity adenoviral vector vascular endothelial growth factorA, FA and ICG were similar. FA showed hyperfluorescent, spotted, roundish areas in the different eyes (**F, H, J, L**). Early and late FA (Figure 2F versus Figure 2H) and ICG (Figure 2G versus Figure 2I) did not show large differences. Early ICG revealed irregularities (black arrow) close to the deeper choroidal blood vessels (**G, K**). These irregularities, shown in Figure 4 and Figure 8C, possibly were caused by alterations and leakage of the choriocapillaris. Late ICG (**I, M**) stained the environment of the spotted roundish areas visible in (**H, L**). Probably, ICG filled areas within the interstitium around cellular proliferations (Figure 4).

**Figure 3:**
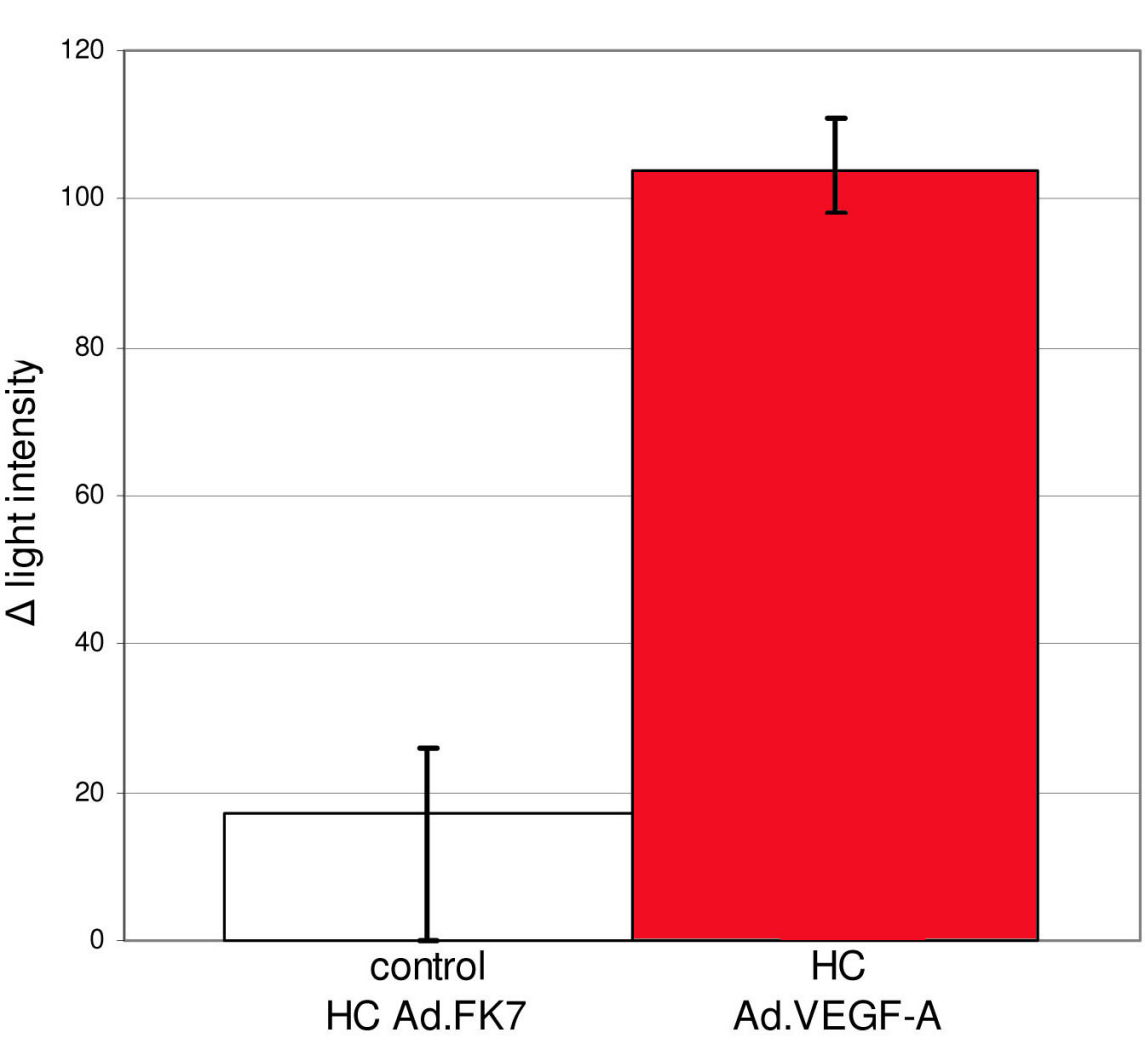
Quantification of fluorescence intensity at the lesion site after fluorescein angiography. The _Δlight intensity_ is the difference between the mean values from light intensity at the lesion site and the mean values from light intensity of an area close to the lesion site. Four weeks postinjection, the Δ_light intensity_ at the lesion site was on average six times higher in eyes that were injected with HC Ad.VEGF-A compared to the eyes that were injected with HC Ad.FK7 (control). The highest/lowest Δ_light intensity_ at the lesion site is indicated by the error bars.

**Figure 4:**
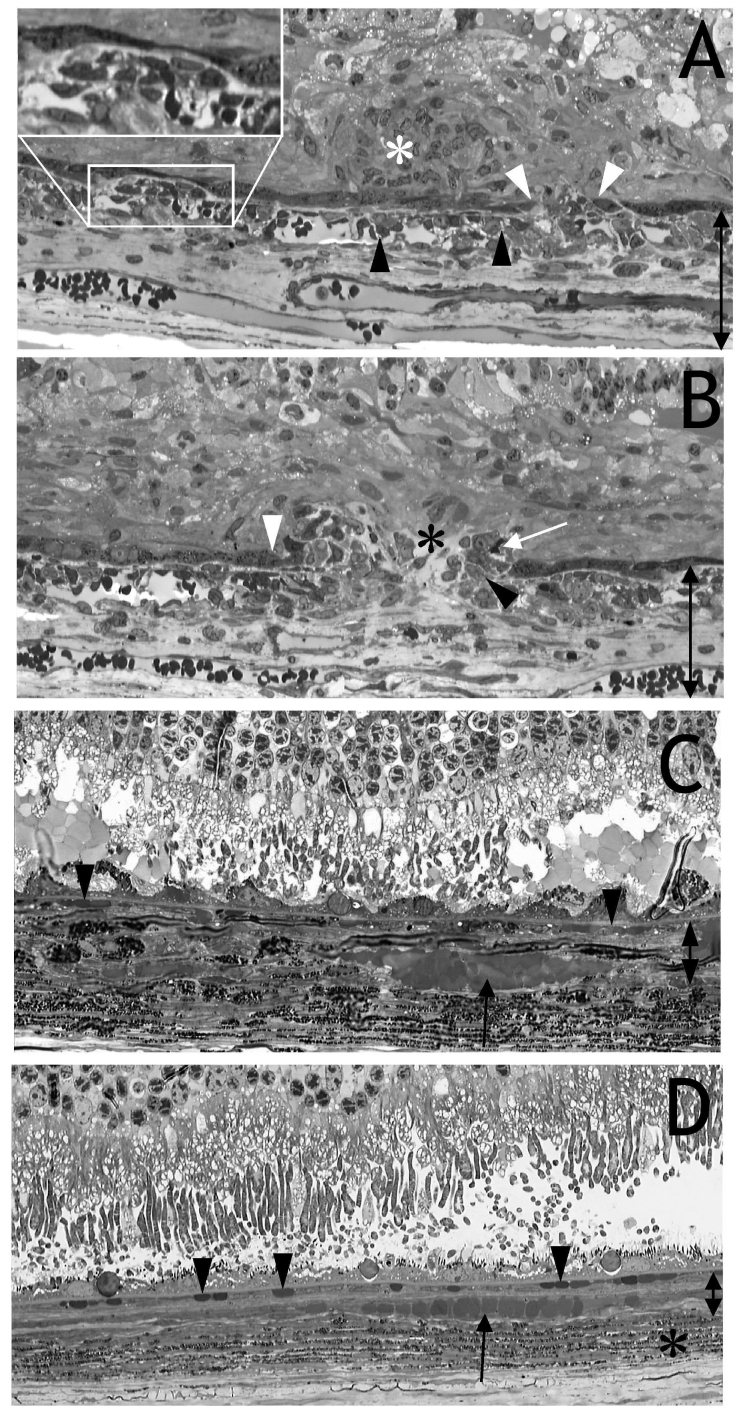
Semithin sections of the eye shown in Figure 2J,K. **A:** The endothelial cell layer of the choriocapillaris was irregular, and endothelial cells protruded into the vessel lumen (black arrowheads). Evidence for this is presented by electron microscopy (see Figure 5A-C). The retinal pigment epithelium (RPE) cell layer was disrupted (white arrowheads), and endothelial cells migrated and proliferated into the subretinal space or between Bruch’s membrane and RPE (inset). Evidence that these cells were endothelial cells is presented by immunohistochemistry (see Figure 6A). These cellular proliferations were either solid (white asterisk in **A**) or loosely packed with interstitial spaces (black asterisk in **B**). The photoreceptors have already degenerated, and retinal scar was closely connected to the RPE and proliferating cells. This was probably why fluorescein leakage was restricted to the spotted roundish areas visible in Figure 2. An immature capillary containing an erythrocyte was located distally to the RPE (**B**, white arrow). The melanocytes of the choroids were located below the deeper choroidal vessels and are not shown. **C** and **D**: After injection of HC Ad. EGFP or PBS, the RPE, choriocapillaris (arrowheads) and deeper choroidal vessels (arrow) appeared to be normal. The pigmented layer, consisting predominately of melanocytes, is marked by a black asterisk. The double arrows in **A-C** indicate growth of extracellular matrix and vessel layers of the choroid after VEGF expression (**A, B**) compared to (**C**).

After transduction with HC Ad.FK7, RPE cells expressed green fluorescent protein (Figure 2A). FA (Figure 2B) or ICG (Figure 2C) did not show blood vessel leakage or abnormalities in any of the four eyes (Table 1). The same was true for the areas in which PBS had been injected subretinally in both eyes (Table 1). No alterations were found after ICG. After transduction with Ad. Empty (Figure 2D,E), which is known to be toxic to the RPE [24], FA (Figure 2D) and ICG (Figure 2E) showed the same image representing a typical window effect. Two weeks (Figure 2F-I) and four weeks (Figure 2J-M) after transduction with HC Ad.VEGF-A, FA and ICG angiographies were similar. However, after transduction with 5x10^6^ iu HC Ad.VEGF-A, FA showed that the areas of injection were hyperfluorescent in 15 out of 18 eyes. These hyperfluorescent areas were either spotty (Figure 2F,H,J,L) or laminary (not shown). Moreover, the quantification of fluorescence intensity at the lesion site after FA revealed that four weeks postinjection, the Δ_light intensity_ was on average six times higher in eyes that were injected with HC Ad. VEGF-A compared to the eyes that were injected with HC Ad.FK7 (Figure 3). Early ICG revealed irregularities close to the deeper choroidal blood vessels (arrow in Figure 2G,K). Late ICG angiography (Figure 2I,M) stained the environment of spotty roundish areas shown in (Figure 2H,L). Within the interstitium around cellular proliferations as shown in Figure 4.

**Table 1:** Overview of the effects of HC Ad. VEGF-A^165^ injection, including control experiments, in rabbit eyes.

**Vector**	**N**	**In vivo observations**	**Ex vivo observations**	
		**Fluorescein leakage in SLO**	**Electron microscopy**	**Immunohisto-chemistry**
HC Ad.VEGF-A	18	15	9	6
PBS	2	0	0	0
HC Ad.FK7 (EGFP)	4	0	0	0
Ad. Empty	4	0	0	0

This table summarizes the experimental procedures employed in this study. The rows indicate the different vectors used (“Vector”; 20 µl subretinal injection at a titer 5x10^6^ i.u. for each vector) and the columns indicate the number of eyes used (“N”) as well as in vivo observations of fluorescein leakage in scanning laser ophthalmoscopy (“fluorescein leakage in SLO”), ex vivo observations at the ultra structural level of neovascularization, endothelial cell proliferation and migration, remodeling of the extracellular matrix, and cellular infiltration of Bruch’s membrane (“Electron microscopy”) and observations in paraffin sections of neovascularization, endothelial cell proliferation and migration, and retinal edema (“Immunohistochemistry”).

### Light microscopy of plastic sections

**Figure 5:**
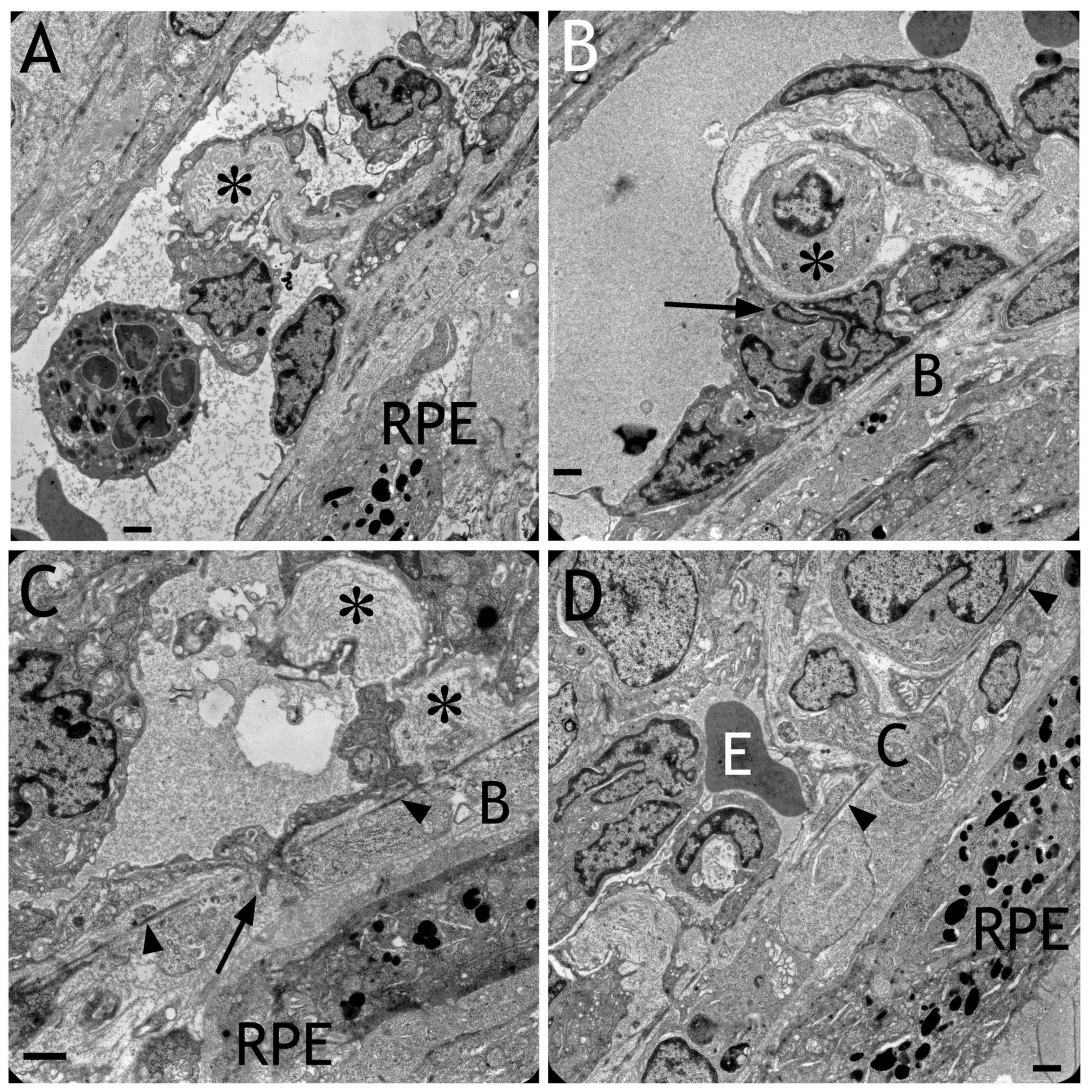
Electron micrographs obtained after transduction with HC Ad.VEGF-A. **A:** An invagination of the the endothelium into the lumen of the choriocapillaris containing extracellular matrix (asterisk) is shown. This caused the patchy appearance of the choriocapillaris lumen presented in Figure 4. **B:** A cell (asterisk) was located between the endothelium and Bruch’s membrane. Note the extremely frayed or fragmented nucleus of an endothelial cell (arrow). **C:** An endothelial cell was spreading into Bruch’s membrane (B) toward the RPE (arrow). The elastic layer of Bruch’s membrane is labeled by arrowheads. Sites indicating remodeling of the extracellular matrix surrounding the choriocapillaris are labeled by asterisks. **D:** A cell (C) was migrating into Bruch’s membrane toward the RPE. The elastic layer of Bruch’s membrane is labeled by arrowheads. Within the pathological capillary, an erythrocyte (E) was still present. Scale bars in each image: 1 μm.

**Figure 6:**
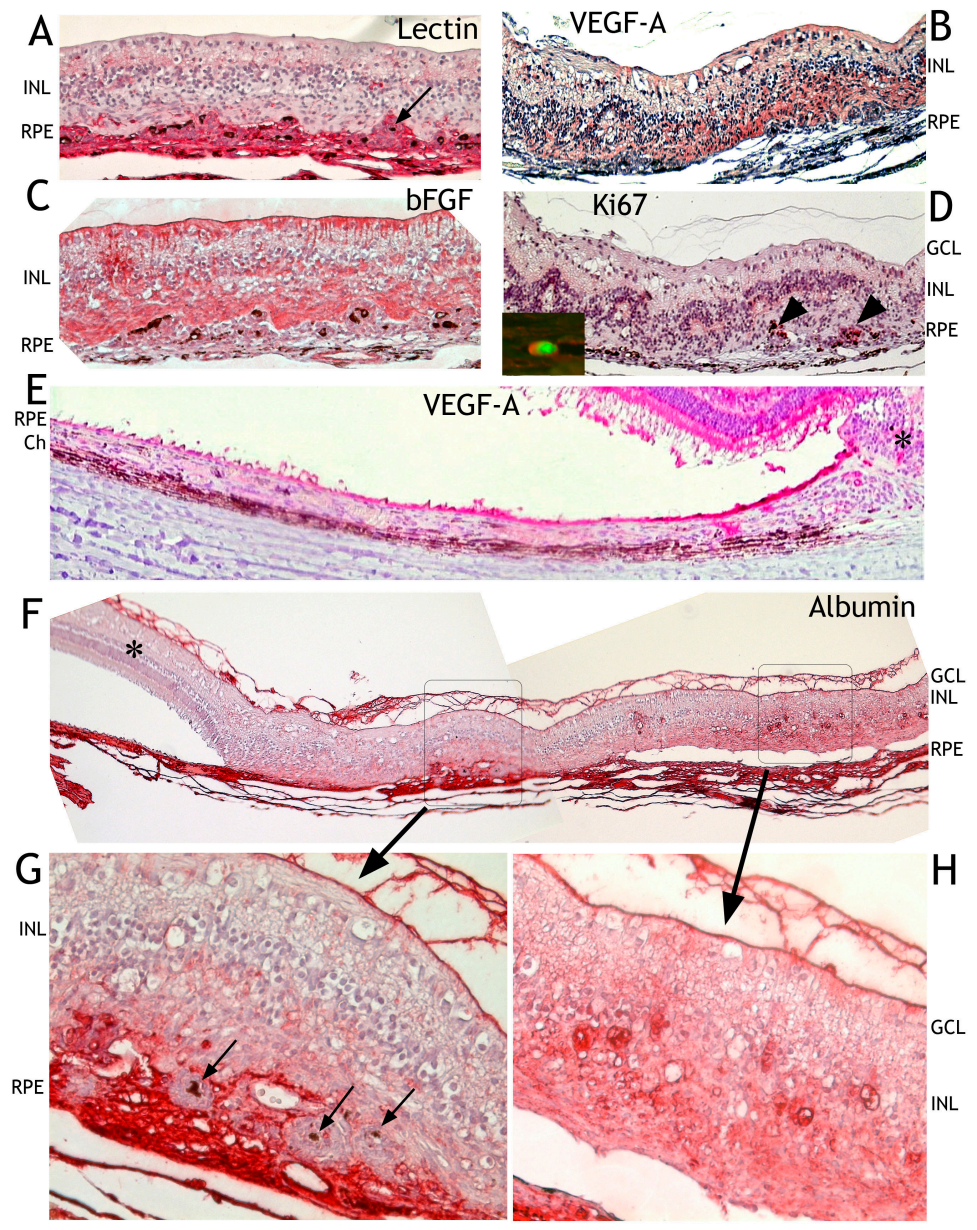
Immunohistochemical findings after transduction with HC Ad.VEGF-A with hematoxylin and eosin counterstain. **A:** Staining with tomato lectin showed that most cells penetrating the disrupted retinal pigment epithelium (RPE) cell layer were of endothelial origin (red). Inner and outer retinal nuclear layers were mixed. Single RPE cells (black arrow) were surrounded by proliferating endothelial cells. **B:** VEGF-A was highly expressed in the retinal scar (white asterisk) and in the RPE close to the retinal scar (black asterisk) but this expression rapidly decreased the farther it was from the retinal scar (**E**). The expression of bFGF was not as strong in cells of choroidal origin than in retinal cells (**C**). **D:** Proliferating cells at the retinal choroidal interface were immunoreactive for Ki67 (black arrowheads). The inset in (**D**) demonstrates the endothelial nature of dividing cells by double labeling for Ki67 (red) and tomato lectin (green). **F:** Albumin (red) was present in the choroid and the fiber matrix at the vitreoretinal interface. It was absent in the unaffected retina (black asterisk). Two selected areas are shown enlarged (**G, H**). Choroidal retinal scarring and leakage of albumin are visible in one of the enlarged areas (**G**). Albumin was not localized within proliferating cells surrounding single RPE cells (black arrows). This may be why ICG did not enter such sites (see Figure 2I,M). **H:** The second enlarged area revealed albumin leakage within the retina that, as shown in this panel, was not fused with RPE or choroid (Ch). Abbreviations: INL, inner nuclear layer; GCL, ganglion cell layer.

**Figure 7:**
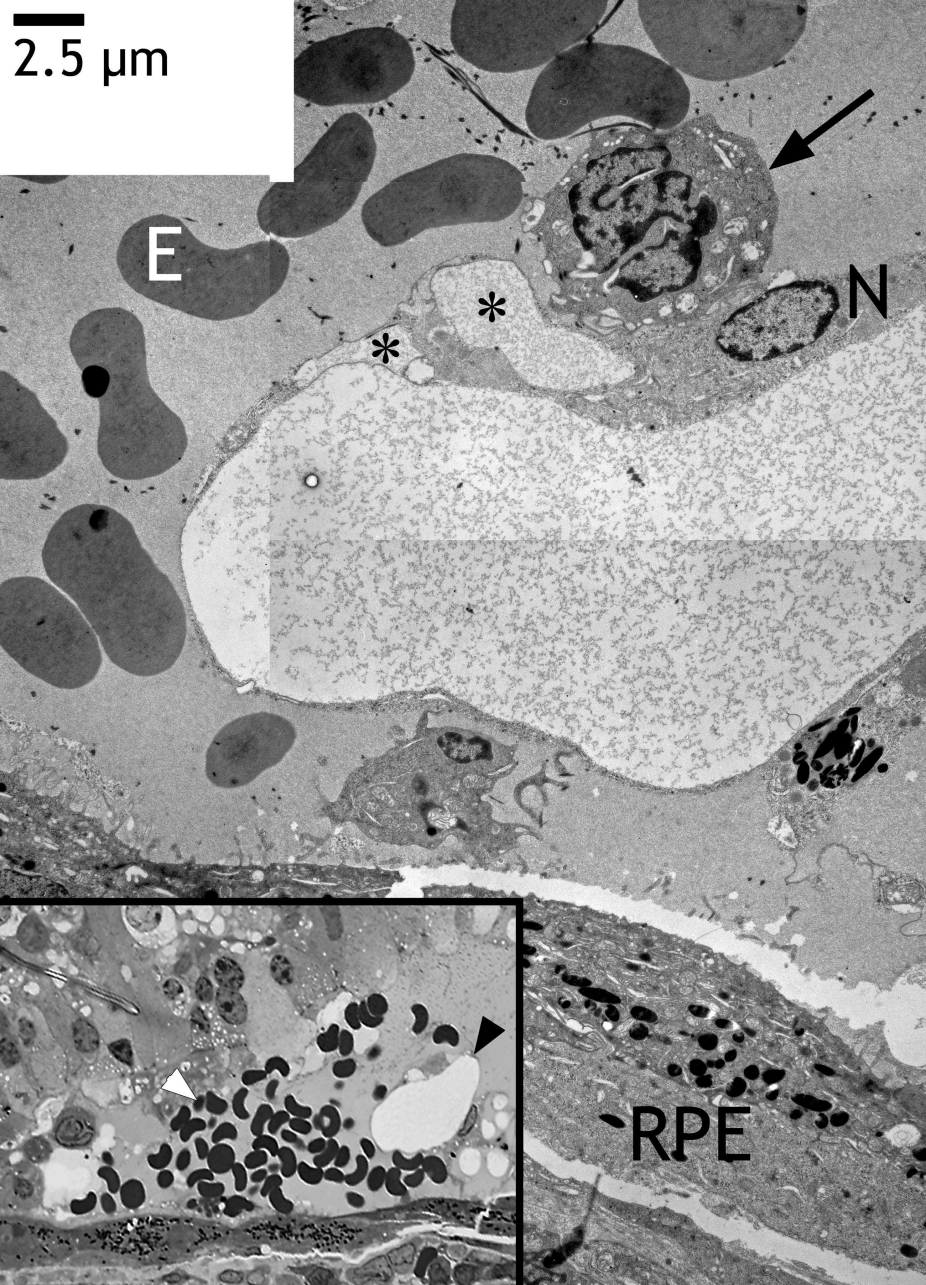
Newly formed capillary in the subretinal space after transduction with HC Ad.VEGF-A from the same eye as shown in Figures 2J,K. The inset (left bottom) shows a semithin section with a new capillary (black arrowhead) within the subretinal space and erythrocytes originating probably from subretinal bleeding (white arrowhead). The photoreceptors of the retina have degenerated and were no longer visible. Electron microscopy revealed the same capillary close to the level of the semithin section. The endothelium was very thin, lacking extracellular matrix. However, it contained vacuoles (asterisks). The endothelium did not contain any fenestrations, and the lumen was free of erythrocytes (E). Note the extremely frayed or fragmented nucleus of an endothelial cell (arrow). The nucleus (N) of the endothelial cell that formed the vessel tube appeared normal. The RPE formed two layers (bottom right).

The following observations were made in all nine eyes investigated at the electron microscopic level and had been injected with 5x10^6^ iu HC Ad.VEGF-A (Table 1). Below the transduced RPE layer, the endothelial cells of the choriocapillaris were irregular, and endothelial cells protruded into the vessel lumen (Figure 4). Evidence for this is presented by electron microscopy (see also Figure 5A-C). The RPE cell layer was frequently disrupted, and endothelial cells migrated and proliferated into the subretinal space or between Bruch’s membrane and the RPE (Figure 4A, inset). Evidence that these cells were endothelial cells was demonstrated by immunohistochemistry (see Figure 6A). Cellular proliferation within the subretinal space was either solid (white asterisk in Figure 4A) or loosely packed with interstitial spaces (black asterisk in Figure 4B). The nuclei of the photoreceptors were mixed with the nuclei of the inner nuclear layer, and a retinal scar was closely connected to the RPE and proliferating cells. This was probably the reason why fluorescein leakage was restricted to the spotty round areas seen in Figure 2D,F. Immature capillaries containing erythrocytes were located distally to the RPE (white arrow in Figure 4B) or in the subretinal space (Figure 7, insert). The thickness of the extracellular matrix and vessel layers of the choroid was three to four times enlarged after transduction with VEGF vector compared to the controls (double arrows in Figure 4A,B). After injection of PBS (in two eyes) or 5x10^6^ iu HC Ad. FK7 (in four eyes), the RPE, the choriocapillaris (arrowheads), and deeper choroidal vessels (arrow), as well as the pigmented layer consisting predominately of melanocytes, appeared normal (Figure 4C,D).

### Immunohistochemistry

After HC Ad.VEGF-A transduction, staining of endothelial cells with tomato lectin showed that most cells penetrating the disrupted RPE cell layer were of endothelial origin. Proliferating endothelial cells surrounded single RPE cells in the subretinal space (Figure 6A). After injection of PBS, only the veins of the retina or the vessels of the choroid with a clear straight borderline to the RPE were stained (not shown).

VEGF-A was highly expressed in transduced RPE cells (Figure 6E). VEGF-A was also strongly expressed in areas with retinal thickening and mixing of inner and outer nuclear layers (Figure 6B). After injection of PBS in the retina, only the inner segments of the photoreceptors and retinal or choroidal vessels showed VEGF immunoreactivity, whereas the RPE was not positive (not shown). bFGF was highly expressed in the scarring retina, particularly in Mueller glial cells and in cells of unknown origin at the interface between retina and choroid. bFGF immunoreactivity was lower in cells of choroidal origin than in retinal cells (Figure 6C). After injection of PBS, only the Mueller cells, the RPE and cells in the choroid showed bFGF immunoreactivity (not shown).

At sites where HC Ad.VEGF-A had been injected, division of cells in the choroid was regularly detected by Ki67 immunoreactivity. Double labeling for Ki67 and tomato lectin indicated the endothelial nature of some of the dividing cells (Figure 6D). After injection of PBS, only a few cells were positive for Ki67 (about 1–2 cells per whole choroid of an eye, not shown).

Albumin immunoreactivity was present in the choroid and the fiber matrix at the vitreal retinal interface. It was absent in the unaffected retina (Figure 6F). In retina close to the choroidal endothelial cell proliferation, albumin was detected within the retina. Albumin immunoreactivity was not localized within the nodular cell cluster surrounding the RPE cells (Figure 6G). This may be why ICG did not enter such sites (see Figure 2G). Albumin leakage within the retina also occurred at sites where the retina was not fused with the RPE or choroid (Figure 6H). After injection of PBS, only choroid and vitreous, but not the retina, stained for albumin (not shown). Single macrophages were regularly detected with CD68 antigen at the injection sites (not shown).

### Electron microscopy

**Figure 8:**
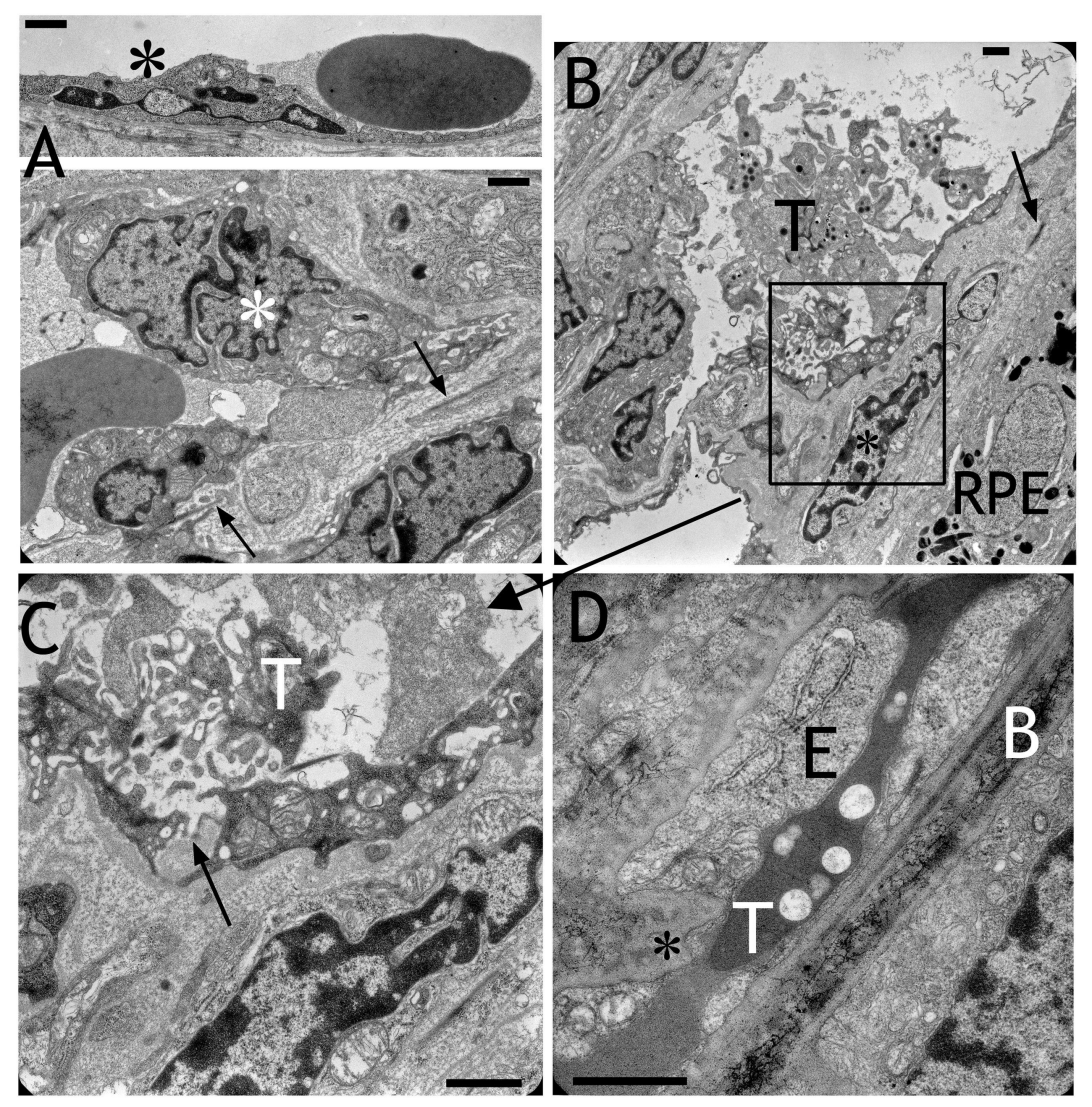
Electron micrographs obtained after transduction with HC Ad.VEGF-A. **A**: Nuclei of the choriocapillaris appeared extremely frayed or fragmented (asterisks). Remnants of the elastic layer of Bruch’s membrane can be recognized (arrows). **B**: Trombocytes (T) aggregated in the choriocapillaris. Cells (black asterisk) migrated into Bruch’s membrane between RPE and choriocapillaris. The elastic layer is marked by an arrow. **C**: Magnification of the frame in (**B**) showed that the adherent junctions were opened and the extracellular matrix had direct contact with the lumen of the capillary (arrow). **D**: The choriocapillaris was completely closed by thrombi (T), which was directly faced Bruch’s membrane (B). The endothelium (E) was thickened and interrupted by another cell (black asterisk). Scale bars in each image equal 1 μm.

After HC Ad.VEGF transduction, nuclei of the choriocapillaris appeared extremely frayed or fragmented (Figure 8A). Thrombocytes aggregated in the choriocapillaris, and cells migrated into Bruch’s membrane between the RPE and choriocapillaris. The adherent junctions were opened, and the extracellular matrix then had direct contact with the lumen of the capillary (Figure 8B,C). Choriocapillaries were frequently completely closed by thrombi. Below the transduced RPE layer, the endothelial cells of the choriocapillaris were irregular and endothelial cells protruded into the vessel lumen as shown in Figure 4. Electron microscopy revealed that this was often caused by invaginations of the endothelium toward the vessel lumen. These invaginations were filled with high amounts of extracellular matrix (Figure 5A). Whole cells, surrounded by extracellular matrix, were located between the endothelium and Bruch’s membrane (Figure 5B). Endothelial cells formed fingerlike protrusions through the elastic layer into Bruch’s membrane (Figure 5C). Sites indicating remodeling and de novo synthesis of the extracellular matrix surrounding the choriocapillaris were always present (Figure 5C). Whole cells were regularly present within Bruch’s membrane (Figure 5D). Newly formed capillaries were detected within the subretinal space (Figure 7). Subretinal bleedings were also present. The endothelium of such capillaries was unusually thin, lacking extracellular matrix but containing vacuoles. The endothelium of such newly formed vessels did not contain any fenestrations, and the lumen was often free of erythrocytes.

## Discussion

Different approaches have been undertaken to induce CNV in rabbits (Table 2), and VEGF was used to induce neovascularization in only three reports [25-27]. However, in just one of these studies, CNV was successfully induced but VEGF was not the principal inducer of neovascularization because matrigel alone had the same effect as matrigel in combination with VEGF [26]. Therefore, the purpose of our study was to develop an efficient and reliable model of CNV to facilitate the study of antiangiogenic and antiproliferative therapies for ocular diseases. This is currently the only model that shows an induction of CNV by overexpression of VEGF in the rabbit. In the present investigation, we observed a CNV with leakage four weeks after the subretinal injection of HC Ad.VEGF-A at 5x10^6^ iu in 83% of the rabbit eyes. This observed leakage did not look like the diffuse leakage observed by patients probably because it was just restricted to the area in which there was an overexpression of VEGF-A–i.e., close to the retinal scar as shown in Figure 6B,E. A window effect as shown in Figure 2D,E was excluded since the RPE was present and the choroidal vessels could not be seen after FA (Figure 2J,L,F,H). We also combined several detection methods of VEGF-A^165^ overexpression induced neovascularization in mammals (SLO, angiography, light microscopy, and electron microscopy). Our findings present clear indications that there is a significant effect on the endothelial cells of the choriocapillaris after a subretinal transduction of the RPE with VEGF-A vector. Moreover, we have described damage of preexisting choriocapillaris and remodeling of extracellular matrix. In accordance with most of the existing rabbit models, we also observed alterations in RPE cells and activation of macrophages that were absent after EGFP transduction (Table 2). The choroidal endothelial cells were activated, adherent junctions opened creating thrombosis and leakage. The fenestration was minimised, while the endothelial cells augment the extracellular matrix and they begin to migrate and infiltrate the Bruch’s membrane. Moreover, they proliferate and form pathological vessels in the subretinal space. We also observed an increased expression of bFGF and VEGF-A accompanied by macrophage stimulation, retinal edema, and photoreceptor loss. In our model, upregulation of VEGF induced a pathological mechanism, in which the choriocapillaris was closed by thrombocyte aggregation, probably inducing hypoxia. Then, hypoxia induced autoregulatory pathways that may have increased or decreased growth factors like bFGF (Figure 6C). This cascade altered the microenvironment leading to CNV. The fact that in laser models, VEGF is secreted or that expression of VEGF induces CNV in specific models does not demonstrate that VEGF plays a major role in the development of CNV in humans with AMD. The conclusions we refer to are that, in normal animal eyes, overexpression of VEGF can induce remodeling of retinal and choroidal vessels and that many of the features of our experimental CNV are similar to those observed clinically in patients having wet AMD (Table 3). Once the CNV is established, it can last for years. Complications, such as retinal and choroidal atrophy, retinal detachment, and RPE cell dysfunction can finally lead to blindness [28]. Indeed, in a previous study by Ni et al. [29], the authors described the time course of experimental CNV induced in rabbits by subretinal injection of endotoxin and bFGF encapsulated into a sustained release format (heparin-sepharose). They demonstrated that the presence of macrophages and the activation of the RPE cells in association with development of CNV suggested an inflammatory component, together with remodeling process, as a major contributor to the development of their model of CNV. They also showed a persistence of clinically leaky CNV lesions throughout the three-year-observation period following a single dose of angiogenic molecules. In most cases, the peak time of CNV leakage appeared at week four [26]. In contrast, laser-induced CNV in rodents and primates has been reported to last usually for just one month. With laser-induced CNV, the peak fluorescein leakage appeared at week two and then gradually resolved because of the rapid maturation of new blood vessels and the reestablishment of the blood-retinal barrier [9]. This is why we chose two and four weeks as time points for our investigations. We did not observe important differences when comparing the angiographies at weeks two or four postoperative indicating that the weak leakage observed at week four was not due to fibrosis and regressing vessels.

**Table 2:** Different methods used to induce CNV in rabbits

**Methods**	**Fluorescein angiography**	**Choroidal neovascularization (CNV)**	**Fenestrations of newly formed vessels detected by EM**	**RPE**	**Inflammation**	**Reference**
Subretinal injection of vitreous	Negative, no leakage	Subretinal vessels in 33%–55% of the eyes after 4–40 weeks	Normal fenestration	Migration; proliferation	n.d.	[34]
Retinotomy with argon laser burns	Not clinically apparent subretinal neovascularization	Microscopical subretinal neovascularization	Normal fenestration	Disruption	Macrophages	[33]
VEGF pellets into the vitreous	Leakage in retinal vessels between 7 and 14 days	n.d.	n.r.;electron micrographs of immature vessels do not show fenetrations	n.d.	n.r.	[25]
Subretinal injection of bFGF micropheres	n.d.	Neovascular channels up to 8 weeks	n.d.	Migration; proliferation	Macrophages appeared early but then disappeared	[30,39]
Subretinal implantation of microsheres impregnated with FGF	CNV leakage in the eyes afer 4 weeks	Subretinal vessels in neovascular membranes	n.d	Migration; proliferation	Macrophages	[40]
Subretinal injection of lipide hydroperoxide	After 100 µg 9%–15% after 4 weeks	Histologically detected in 46% of the eyes	Normal fenestration	Detachment, damage	Macrophages	[32]
Subretinal injection of FGF-2 and LPS in Heparin-Sepharose beads	CNV leakage in 100% of the eyes afer 2 weeks and 3 months	CNV in all injectied eyes	n.d	Proliferation	Relationship of Makrophages to CNV	[29]
Subretinal injection of matrigel alone or in combination with VEGF	CNV leakage in 100% of the eyes afer 2 - 4 weeks also without VEGF	CNV in 100% of the eyes afer 2 - 4 weeks also without VEGF	n.d.	Displacement, vacuolisation, depigmentation	Many inflammatory cells	[26]
Injection of AdVEGF-A into the vitreous	n.d.	n.r.	n.d.	n.r.	Strong inflammatory reaction	[27]
Subretinal injection of HC-AdVEGF-A	CNV leakage in 83% of the eyes after 2–4 weeks (5x10^6^ i.u.)	Observed after 2–4 weeks	Not found in newly formed vessels	Migration; proliferation	Few macrophages	Present study

This table summarizes the data from the literature concerning the different methods of CNV induction in rabbits. Abbreviations: n.d.=not determined, n.r.=not reported.

**Table 3:** Similitude of our experimental rabbit CNV model with the features observed in patients suffering from wet AMD

-> Changes in the RPE (hypo- or hyperpigmentation)
-> Bruch’s membrane loss and infiltration of endothelial cells
-> Remodelling of extracellular matrix
-> Subretinal neovascularisation
-> Proliferation of endothelial cells and fibroblasts
-> Subretinal haemorrhage
-> Leakages
-> Thrombosis
-> Loss of photoreceptors
-> Retinal edema
-> Upregulation of bFGF and VEGF

The current study is the first in which a direct effect on the choroidal endothelial cells after transduction with a VEGF vector in rabbits has been proven. Although Matrigel was subretinally injected in combination with VEGF in an earlier study [26], the VEGF had no effect there, and the subretinal injection of Matrigel alone showed the same effect on the choroidal blood vessels. In this study, introducing gel-like Matrigel compounds into the subretinal space created a physical barrier between the neural retina and its underlying choroid tissues. Therefore, the mechanism leading to CNV seems to be different from AMD, where basal laminar deposits are located in Bruch’s membrane between RPE and choroid.

In a 2006 study done by Kinnunen et al. [27], a VEGF-A adenovirus was injected into the vitreous body. Production of VEGF, endothelial cell proliferation, as well as increased capillary density in the retina, the optic nerve head, and the anterior segments were also detected. However, the effect on the choroidal vessels was not investigated.

Other investigators have reported using angiogenic molecules to induce CNV in rabbits [25,30-34]. Tamai et al. [32] reported fluorescein leakage after four weeks in 9%–15% of rabbit eyes using subretinal injection of lipid hydroperoxide (100 μg), whereas CNV formation was found in up to 46% of rabbit eyes. They also performed light and transmission electron microscopy, which showed a degeneration of the retina accompanied by a detachment of RPE, and the presence of macrophages. They also described a normal fenestration of newly formed vessels detected by electron microscopy. Investigators also attempted to stimulate CNV in rabbits through intravitreal VEGF administration [25]; however, only a transient (three weeks) retinal neovascularization was observed following intravitreous implantation of VEGF pellets. In a 1991 study [33], the authors tried to create CNV by performing retinotomy with argon laser burns. Although they did not observe clinically apparent CNV, all laser lesions contained microscopic CNV. This model was also associated with a degeneration of the retina, the disruption of RPE, and the presence of macrophages. The first described model of experimental subretinal neovascularization in the rabbit was performed in 1989 and was induced by subretinal injection of vitreous without rupture of Bruch’s membrane [34]. The incidence of CNV rose from 33% to 57% in a period of 4–40 weeks. Because of the absence of any fluorescein angiographic indication of CNV, these occult new vessels were identified by light and transmission electron microscopy. Histological examination showed that these newly formed vessels were composed of continuous capillaries and had the morphologic characteristics of choriocapillaris, including fenestrations, basement membranes, and junctional complexes. The new vessels originated from the choriocapillaris and penetrated through Bruch’s membrane into the subretinal space, where they were associated with the degenerated retina and proliferating glial and/or RPE cells.

To conclude, our rabbit model for CNV offers the possibility to define not only the molecular signals involved but also to examine how they interact and how the specific microenvironments in the eye influence neovascularization. It will also provide additional new targets for intervention and allow testing of drugs that block the targets. Inhibitors of VEGF signaling have already been demonstrated to be useful for prevention of retinal neovascularization or CNV in animal models and in patients [35-38]. A major goal for the future is to precisely identify and test precisely new agents or combination of agents that cause regression of neovascularization in a rabbit model like ours under exactly defined conditions.
